# Consumer Motivation in Developed Economies With Secular Stagnation

**DOI:** 10.3389/fpsyg.2019.02697

**Published:** 2019-12-02

**Authors:** Fernando Evaristo Callejas-Albiñana, Irene Martín de Vidales Carrasco, Isabel Martínez-Rodríguez, Ana Isabel Callejas-Albiñana

**Affiliations:** ^1^Department of Spanish and International Economy, Econometrics, History and Economic Institutions, University of Castilla–La Mancha, Ciudad Real, Spain; ^2^Department of Political Economy and Public Finance, Economic Statistics, Business and Economic Policy, University of Castilla–La Mancha, Ciudad Real, Spain; ^3^Department of Psychology, University of Castilla–La Mancha, Ciudad Real, Spain

**Keywords:** secular stagnation, consumer motivation, financial cycle, technological innovation, social inequality

## Abstract

In recent years, the slow pace of economic growth, high indebtedness, and high unemployment registered in most developed economies since 2009 have revived the debate over the “secular stagnation hypothesis” first formulated by the Keynesian economist Alvin Hansen in 1938. This return of the secular stagnation hypothesis occurred in November 2013, when Lawrence Summers postulated that the global economy was facing a scenario of low growth, low inflation, and a reduction in GDP per capita due to a chronic insufficiency of aggregate demand. The causes should be sought not only in cyclical factors associated with a long financial cycle and excessive accumulated public and private debt, but also in structural changes in the central economies in recent decades, linked to the rapid slowdown in population growth and the gradual aging of the population. Finally, other factors also depress demand, such as the progressive exhaustion of the globalization process and the consolidation of new labor models. In light of these developments, this paper’s aim is twofold: first, to perform an econometric panel-data study in order to determine the influence of each of these factors in explaining secular stagnation in recent years for the selected sample of countries; and, second, to lay out proposals for reorienting the government intervention strategies adopted since the onset of the financial crisis to promote consumption and achieve sustained growth, job creation, and poverty reduction.

## Introduction

In November 2013, Lawrence Summers revived the “secular stagnation hypothesis” first formulated by Keynesian economist Alvin Hansen in 1938. In doing so, he initiated an interesting debate on the state of developed country economies after the Great Recession, asserting that the global economy faced a scenario of low growth, low inflation, and a reduction in per capita GDP due to chronic insufficiency of aggregate demand.

This scenario is the starting point for the present research, which has a twofold objective. The first is to identify the short-term and structural factors that explain secular stagnation and perform an econometric panel-data study to analyze the influence of each one in recent years for the selected sample of countries. The second is to lay out proposals to reorient the government intervention strategies adopted since the onset of the financial crisis to promote sustained growth, job creation, and poverty reduction. To achieve these goals, one strategy to be taken into account is that of examining motivations for entrepreneurship, not only in terms of how the internal or external incentives provided by companies might contribute, as discussed in the classic organizational psychology theories of McClelland (1961) and Herzberg (1970), but also because of the interest of producing entrepreneurs to contribute to economic growth and job creation.

## Materials and Methods

### The Secular Stagnation Hypothesis: Origins and Explanatory Factors

The secular stagnation hypothesis was first formulated by the economist and Keynes disciple Alvin Harvey Hansen (1887–1975) in his famous speech to the American Economic Association at its annual meeting in December 1938.^1^ Hansen argued that, due to certain structural characteristics (such as the declining population growth rate and scant technological advances), economies tended to show a greater propensity to save than to invest, slowing down economic growth (Sandoval and Morales, 2017: 58).

Precisely 1 year earlier, in 1937, the American economy had entered into a strong recession following a period of dynamism achieved through the implementation of the New Deal. Franklin Delano Roosevelt had adopted that plan in the spring of 1933 to overcome the serious effects of the Great Depression of 1929 (spectacular increase in unemployment, contraction of international trade, decline in wages, and a notable loss of purchasing power).

In keeping with the postulates of Keynesian theory, the New Deal defended the need for government intervention in the economy, that is, for the mobilization of public money to generate economic activity and purchasing power. It had two main dimensions, economic and social (Lozano, 2004; Serrano, 2010: 25–128).

(a) The *economic dimension* addressed the major problems in three key sectors:
  • The financial system, by increasing government control over banking institutions in order to ensure their solvency, stimulates the granting of credits for business investment, and protects investors from possible fraud. In particular, two important regulations were enacted to restore confidence in the financial system:
    ˚ “*The Emergency Banking Act*” (March 9, 1933) formulated a program to clean up the banking sector by which only those entities that proved to be sufficiently solvent were allowed to operate, granting them a guarantee from the Federal Reserve that insured 100% of bank deposits (Federal Reserve, 2019b).
    ˚ “*The Banking Act*” (June 16, 1933), better known as the *Glass Steagall Act*, created the Federal Deposit Insurance Corporation; consolidated the limitations on branch opening regulated in the *McFadden Act* of 1927; prohibited commercial banking from participating in the investment banking business; and provided for the elimination of interest on bank deposits (Federal Reserve, 2019a).
  • Industry, by encouraging subsidies to industry to encourage its recovery (“*National Industrial Recovery Act*” of June 16, 1933), in addition to the undertaking of gigantic public works projects with a view to industrializing certain areas of the country (mainly the Tennessee River Valley) and creating millions of jobs.
  • The agricultural sector, through the granting of compensation, with the primary aim of reducing the overproduction that had caused the prices of agricultural products and farmers’ profits to plunge (“*Agricultural Adjustment Act*” of May 12, 1933).
(b) The social dimension encompassed intervention in two major areas:
  • Labor, by regulating employer–worker relations, the minimum wage, and maximum working hours (“*National Labor Relations Act*” of July 6, 1935).
  • Welfare, by promoting the creation of the first federal system of unemployment insurance and pensions, aimed at correcting the considerable existing social inequalities (“*Social Security Act*” of August 14, 1935).

However, as noted, most of the gains made during the economic recovery process beginning in 1934 were lost in late 1937, when the Roosevelt administration began to gradually reduce public spending and rein its expansionary monetary policies. The precarious state of the economy became apparent when, as these government support measures were withdrawn, unemployment rose from 14 to 19%, affecting >7 million citizens, industrial production fell by 37%, and family incomes fell by 15% (Jones, 1980; Redondo, 2015). That was when Hansen put forward his hypothesis that there had not been a normal economic cycle, but rather a depression that could become “secular” if decisive action were not taken.

Indeed, this Keynesian economist claimed that the reduced possibilities of internal geographic expansion, the observed decline in population growth (probably due to the severity of the crisis), and the tendency for new technologies to use less capital than in the early stages of capitalist development would explain the inability of investment to generate the demand that would ensure full employment and the corresponding real output. As a result, savings would accumulate unused, and economic growth would plummet unless governments borrowed and spent to reactivate aggregate demand.

However, Hansen qualified his thesis, considering that the real cause of economic stagnation was the scarcity of profitable investment opportunities compared to earlier periods. Therefore, the solution would not be simply to stimulate demand through public spending and cheap financing. In other words, it was not only a problem of demand, but also supply, which the private sector alone was not capable of solving (Navascués, 2016).

In short, according to his theory, demand had to be stimulated, but very selectively, to prevent any public spending from being diverted to superfluous objectives. To this end, nothing could be better than a public investment program that would generate positive side-effects for consumer spending and private investment, with a view, in particular, to improving the standard of living of low-income social groups. At the same time, this spending program would have to be combined with a flexible tax program, allowing the basic rates on revenue to be adjusted within the limits set by the government, i.e., the adoption of a compensatory fiscal policy, enabling a corrective synchronization of public spending and taxes with the economic cycle (Antonelli, 1957).

Additionally, the supply side shortcomings would have to be corrected by improving education and training and promoting technological innovation. However, Hansen was quite skeptical of the government’s management capacity, arguing, “We have not yet learned to make the Government an effective, flexible and sensitive instrument in a fluctuating and highly complex society, in a private enterprise society in which the State must take on the role not only of counterweight, compensating for the shortcomings of private investment, but also as a provider of very important public services and as an advocate of basic development programs” (Antonelli, 1957: 24).

The truth is that the economic recession that began in 1937 was short-lived, thanks largely to the immediate and forceful response of the Roosevelt administration. Disregarding the Treasury Department’s recommendations to continue the efforts to balance the budget, it launched a $5 billion spending program in the spring of 1938 to increase purchasing power.

This change in the orientation of the economic policy measures implemented enabled the rapid recovery of industrial production, which grew again to 30%. The economic reactivation was also later linked to the expenses generated by the Second World War, which was an important stimulus factor and kept the level of activity very high.

So, time proved Hansen profoundly wrong, not only because of the fairly high birth rate of the baby boomers, but above all because of the high investment accompanied by unprecedented global growth.^2^ The secular stagnation hypothesis thus fell into oblivion for a long period of time.

### The Current Economic Debate on Secular Stagnation

However, in recent years, the slow pace of economic growth, the high level of indebtedness, and the high unemployment registered in most developed economies since 2009, due to both the serious damage caused by the Great Recession and structural changes the economy has been facing for several decades, have revived the debate over the secular stagnation hypothesis, in both the financial press and mainstream academic economics circles.

Indeed, in the summer of 2007, the global economy entered into a major financial crisis, including the collapse of the housing market, a sharp fall in asset prices, deleveraging of the domestic and financial sector, strict credit conditions, and a high degree of political uncertainty and instability. This state of affairs subsequently triggered a severe economic recession, indeed, the most intense, problematic, and prolonged of any experienced in the last 80 years. The crisis was especially harsh for the countries on the periphery of the euro zone, due to the excessive accumulated debt (Costas and Arias, 2015; Nash-Stacey and Karp, 2015).

Nevertheless, as noted, the fragile economic situation that has prevailed in industrialized countries since 2009 is not due solely to the effects of the recent systemic financial crisis and the persistence of large volumes of public and private off-balance-sheet debt. It is also due to structural changes that have been underway since 1980, such as the slowdown in population growth, the falling price of capital goods, and increasing income concentration, which hinder sustained long-term growth, i.e., a rate of economic growth compatible with full employment. The combined action of all these factors (cyclical and structural) results in excess savings over aggregate investment, which ultimately leads to a stagnation in economic activity, accompanied by strong deflationary tendencies (Pérez and Delbianco, 2015: 53).

Therefore, the return of the secular stagnation hypothesis in late 2013, at the hands of Summers, a Harvard economist and former U.S. Treasury Secretary under President Bill Clinton, took place in a context and in conditions similar to those that Hansen and other authors of the Keynesian school tried to explain and understand eight decades ago.

Speaking at the November 2013 International Monetary Fund (IMF) academic conference in honor of Stanley Fischer, Summers hypothesized that the world economy was in a situation of “secular stagnation,” launching an interesting debate on the state of developed country economies after the Great Recession.^3^

According to his thesis, in their current structural configuration, central economies are incapable of simultaneously achieving satisfactory economic growth and minimum conditions of financial stability (Summers, 2014a: 29). In other words, the global economy is facing a scenario of low growth, low inflation, and a reduction in per capita GDP due to a chronic insufficiency of aggregate demand. The most visible manifestation of this phenomenon is the downward trend in interest rates, reflecting a greater propensity to save than to invest, which inevitably translates to a loss of economic dynamism. In addition, the term “secular” used to qualify this situation implies a process of indefinite duration or, at least, a very long stagnation (Quirós, 2015).

This structural imbalance between aggregate savings and investment gives rise to the chronic insufficiency of demand and, thus, provides support for the secular stagnation hypothesis. It is basically due to the following long-term changes registered in central economies in recent decades (Pérez, 2015):

(a) The rapid *slowdown in population growth* since 1960, in the United States, Europe, and Japan, which has logically been accompanied by a reduction in the workforce. This, in turn, has had a negative impact on capital accumulation.
  In addition, the notable change in the *population’s age structure*, due to the declining birth rate and higher life expectancy, is a very important factor that could be driving savings up in the world, to the detriment of consumption, and, consequently, aggregate demand down. Specifically, the last 30 years have seen a significant increase in the size of the population group between the ages of 45 and 64 years. This group’s economic behavior is strongly conditioned by the need to maintain a certain standard of living after retirement and it thus tends to save more. The current existence of very weak or even non-existent public or pay-as-you-go pension systems must also be taken into account in this regard.
  In short, demographic variables are clearly hampering aggregate demand, not only because of lower population growth in the main developed countries, but also because of the significant expansion of the population strata that are more likely to save (Martí, 2017a).
(b) The *fall in total factor productivity* (TFP)^4^ caused by a paralysis in technological innovation, especially following the onset of the financial crisis in 2007, and/or because new information technologies and digitization do not have as significant an impact on real economic activity as the manufacturing technologies of the twentieth century did (e.g., internal combustion, electricity, automobiles, aviation, telephony, television, lasers, the Internet, or mobile telephony).
  In contrast to this “pessimism” regarding future technological progress, highlighted by neo-Keynesian economists such as Gordon (2012),^5^ other authors, such as the University of California economic historian Eichengreen (2014) and Eichengreen et al. (2015), argue that the reasons for the stagnation of production and total productivity should be sought in the failure of some developed countries, such as the United States, to invest in infrastructure, education, and training, rather than in the scarcity of potentially revolutionary innovations.^6^ In other words, to achieve sustained economic growth, it is not enough to increase investment in R&D; it is also necessary to ensure that the economy is capable of reallocating production factors to their most efficient uses.
  Additionally, one widespread argument notes that the service sector, where productivity growth is significantly slower than in the industrial sector, tends to account for an increasing share of advanced economies, in terms of both real output and job creation (Bach, 2016).
(c) The *growing inequality in income and wealth distribution*^7^ is another key explanatory factor for the greater aggregate marginal propensity to save witnessed in developed countries in recent years. Indeed, because the economic actors with the highest incomes are more likely than the poorest ones to save money rather than spend it, increasing inequality will dampen consumption, the financial position of the middle class, and, ultimately, the growth of aggregate demand (Guillén and Ontiveros, 2014).
  Summers also recognized that this structural imbalance between aggregate savings and investment is further explained by the increase in gross corporate savings, due to the rising share of profits in the income of major industrialized countries since the mid-1970s and early 1980s. In particular, this is the result of rising globalization since 1980 and the increase in technological progress over this period (Summers, 2014b).
  Although they do not explicitly adhere to the secular stagnation thesis, other authors, including the French economist Thomas Piketty, argue that the strong increase in social inequality registered in developed countries over the last three decades, with the large-scale implementation of free-market postulates and financial deregulation, will inevitably translate to continued low economic growth accompanied by low population growth in the coming decades (Piketty, 2015).
(d) *Other factors depressing aggregate demand*, including the *gradual exhaustion of the globalization process*, which, after accelerating dramatically over the last 20 years, especially as a result of the Uruguay Round of the GATT-WTO^8^ and, above all, China’s entry into the Organization on December 11, 2001,^9^ has come to a virtual halt. As a consequence, the world stock of productive capital is already largely adapted to the current scheme of comparative and competitive advantages in the international trade system. This will probably be associated with lower rates of economic growth due to the progressive exhaustion of this source of generation of new investments (Martí, 2018).

Finally, another factor that could currently be contributing decisively to the increase in savings worldwide and, therefore, to the stagnation of aggregate demand is the *consolidation of new employment models*. These models are largely characterized by less employment stability, i.e., an erosion of permanent employment coupled with a strong trend toward temporary employment. These temporary contracts are being used by entrepreneurs not only to cover companies’ short-term needs, but also as an instrument for providing external and internal flexibility to their productive organizations.

The intensely precarious nature of this new employment (the so-called “gig economy”) has had a clearly negative impact on public social security systems and, more specifically, on unemployment protection systems, unbalancing them financially. One immediate consequence of the greater labor risks assumed by a good number of workers would be an increase in the level of savings, in order to cope with any contingencies that might arise and, thus, weaker consumption. This behavior might also be more likely to manifest among workers with higher salaries, since, in their case, public sector insurance would cover a smaller part of their previous labor income (Cavas-Martínez, 2005; Martí, 2017b).

In short, although the international economic situation has improved comparatively in recent years, and has done so quite generally across countries and geographical areas, all these structural factors, coupled with the more typically short-term factors associated with the effects of the recent global financial crisis and the previously high levels of indebtedness (Koo, 2014), have considerably reduced domestic and corporate investment, significantly weakening aggregate demand (Table 1).

**TABLE 1:** Explanatory factors of secular stagnation.

**Short-term factors**	**Structural factors**
• Long-term *financial cycle*, accompanied by banking crises and growing tensions in global financial markets.	• Demographic factors: ✓Rapid slowdown in world population growth. ✓Gradual aging of the world population.
	• Declining TFP
• Excessive accumulated *public and private debt* (balance sheet recession).	• Increasing *inequality in income* and *wealth distribution*
	• Other factors depressing *aggregate demand*:
	✓Gradual exhaustion of the globalization process.
	✓Consolidation of new employment models: less job stability.

On the other hand, in recent years many economists have defended the effectiveness of actions on aggregate supply to increase the productive potential of economies and achieve sustainable long-term growth. Thus, as mentioned at the beginning, it is possible to mitigate the current weakness of aggregate demand by considering the relevance of personal factors of entrepreneurship, as the motivation of achievement, being a characteristic of the personality that allows to relate with the success of the created companies (Barba and Atienza, 2011).

As noted at the start of this paper, certain factors related to individual entrepreneurship can partially mitigate this effect, such as the motivation of achievement characteristic of personalities associated with the success of created companies (Barba and Atienza, 2011).

In this context, Shapero (1985) considers that the decision to start a company depends on two types of personal perceptions having to do with values related to culture, socioeconomic status, family, education, and influential people: the *perceived feasibility* and the *perceived desirability* of starting one.

Many theories of human motivation have sought to identify the factors that define the entrepreneurial behavior that improves a country’s economy and that drive successful behaviors in this regard, using the personality of entrepreneurs as a source. For example, Paturel (1997) identifies three factors: the future entrepreneur’s aspirations, his or her skills and resources, and the opportunities afforded by the market.

These types of incentivized strategies (to encourage entrepreneurs) work on several fronts: the decline in productivity, which they slow, albeit moderately, since the entrepreneur him or herself is the beneficiary of the work’s results; supply (direct for this worker profile); and demand, since rental income increases and so, thus, will consumption.

## Results

### Empirical Analysis

The main objective of this empirical study is to determine which factors explain a possible secular stagnation of the economy. To this end, the impact of such factors on economic growth is quantified using the statistical method of panel data analysis^10^ with a sample of 12 countries (sufficient statistical information) around the world^11^ and time series from 1998 to 2017.

An econometric panel-data model includes a sample of economic agents or stakeholders (individuals, firms, banks, cities, countries, etc.) for a given period of time, i.e., it combines both short-term and structural data (Mayorga and Muñoz, 2000). Specifically, here this model is used to study the impact of a set of short-term and structural variables on the economic growth of the 12 selected countries. The countries selected for the model lend themselves to the fixed-effects estimation method.

With panel sectional time series data, the most commonly estimated models are probably fixed effects (FEs) and random effects models, so is important to establish which of them is the most adequate.^12^ Several considerations affect the choice between a FE and a random effects model (see Allison, 2009) but, in this case three points have been considered.

Firstly, the nature of the variables that have been omitted from the model are correlated with the variables in the model so, FE models may provide a means for controlling for omitted variable bias. Secondly, the subjects (countries, in this case) change across time and there is within-subject variability in the variables, so a FE model is more convenient. Thirdly, the subjects are not randomly selected, but they are selected with a specific criterion. Specifically, those considered as world powers by the World Bank in 2017 (by registering a higher level of GDP per capita). So, the preliminary choice of countries determines that the FEs be the most appropriate method.

In short, for all that, the estimation must be made with FE.

The variables were selected to represent the short-term and structural factors explaining secular stagnation shown in Table 1. In all, 18 variables were selected (Supplementary Table 1):

• *Variables representing short-term factors*. Initially, a total of four variables representing the financial cycle and excessive public and private debt were considered, of which two were significant for the final models: stocks traded (total value as% of GDP)^13^ and total debt (current euros) (Table 2).
• *Variables representing structural factors*. Initially 10 variables were considered, representing demographic factors, factors depressing aggregate demand, growing inequality in income and wealth distribution, and TFP. Of these, five were ultimately significant for the final models: total unemployment (% of total workforce), population aged 65 years or over (% of total), TFP growth (original), TFP growth (adjusted), and TFP at current prices (USA = 1) (Table 3).
• *Variables representing economic growth* (*and thus secular stagnation*). Initially, a total of four representative variables were considered, of which three were ultimately significant for the final models: GDP growth (annual %), GDP growth per capita (annual %), and GDP per capita (US$ at current prices) (Table 4).

**TABLE 2:** Variables corresponding to the short-term factors explaining secular stagnation.

**Indicator**	**Factor**	**Database**	**Acronym**
Stocks traded, total value (% of GDP)	Financial cycle	World Federation of Exchanges database	ST_P
Stocks traded, total value (current US$)	Financial cycle	World Federation of Exchanges database	ST
Total debt	Excessive debt	Datosmacro.com	TD
Total debt (per capita)	Excessive debt	Datosmacro.com	TD_PC

**TABLE 3:** Variables corresponding to the structural factors explaining secular stagnation.

**Indicator**	**Factor**	**Database**	**Acronym**
Unemployment, total (% of total workforce)	Aggregate demand	International Labour Organization, ILOSTAT database (updated 2017)	UNE
Gini index	Inequality	World Bank, Development Research Group	GINI
Population growth (annual %)	Demographics	(1) United Nations Population Division: World Population Prospects; (2) Census reports and other statistical publications by national statistics bureaus; (3) Eurostat: Demographic Statistics; (4) United Nations Statistical Division: Population and Vital Statistics Report (various years); (5) U.S. Census Bureau: International Database; and (6) Secretariat of the Pacific Community: Statistics and Demography Programme.	PG_P
Population aged 65 years and over (total)	Demographics	World Bank. Based on the United Nations Population Division’s World Population Prospects age/sex distribution (updated 2017)	P65
Population aged 65 years and over (% of total)			P65_P
Population aged 0–14 (% of total)			P14
TFP at current prices (USA = 1)	TFP	Penn World Table, version 9.0^a^	CTFP
TFP at constant national prices (2011 = 1)			RTFPNA
TFP growth (adjusted)		The Conference Board (TCB). Total Economy Database.	TFPA
TFP growth (original)			TFPO

*^*a*^Feenstra et al. (2015).*

**TABLE 4:** Variables corresponding to indicators of secular stagnation.

**Indicator**	**Database**	**Acronym**
GDP growth (annual %)	World Bank national accounts data and OECD national accounts data	GDP_P
GDP growth per capita (annual %)		GDP_PCP
GDP per capita (current US$)		GDP_PC_N
GDP per capita (constant local currency)		GDP_PC_R

As seen in the correlation matrix (Supplementary Dataset 1), there is no serious multicollinearity among the independent variables because the most variables correlate from very slight to moderate (0.004 ≤ *r* ≥ 0.5). This indicates that the different measurement scale of the independent variables does not affect the relation among them. However, following the guidelines by Afifi et al. (2012), the indicators with a correlation >0.5 are not included in the same econometric model.

The regression model results (Supplementary Dataset 2) by using panel data estimation are shown in Table 5. These models suggest that it is valid (*P* = 0.05 or *P* = 0.01) to study and analyze GDP (measured by GDP growth, GDP growth per capita, and GDP per capita) through the structural and short-term factors considered (Tables 2, 3).

**TABLE 5:** Regression results: estimation with panel data.

**Variable**			**Dependent variable**		
			
			**GDP growth (annual %)**	**GDP growth per capita (annual %)**	**GDP per capita (US$ at current prices)**
			
			**Standardized coefficients (Student’s *t*-values)**		
			
		**^(1)^**	**Model 1: GDP_P**	**Model 2: GDP_PCP**	**Model 3: GDP_PC_N**
Stocks traded, total value (% of GDP)	(ST_P)	(SH)			0.118 (2.62)^∗∗^
Total debt	(TD)	(SH)		0.159(2.23)^∗∗^	
Unemployment rate	(UNE)	(ST)	−0.312 (−7.27)^∗∗∗^	−0.206 (−4.30)^∗∗∗^	−0.123 (−2.59)^∗∗^
Population aged 65 years and over (% of total)	(P65_P)	(ST)	−0.273 (−3.28)^∗∗∗^	−0.418 (−3.85)^∗∗∗^	
TFP at current prices (USA = 1)	(CTFP)	(ST)			−1.045 (−9.96)^∗∗∗^
Total productivity growth (adjusted factors)	(TFPA)	(ST)		0.743 (19.86)^∗∗∗^	
Total productivity growth (original factors)	(TFPO)	(ST)	0.739 (23.19)^∗∗∗^		
Statistical models			*n* = 228 *F* = 200.18^∗∗∗^ *R*^2^ set = 0.78	*n* = 219 *F* = 108.71^∗∗∗^ *R*^2^ set = 0.73	*n* = 196 *F* = 34.33^∗∗∗^ *R*^2^ set = 0.49

*(1) Factor the variable represents: (SH), short-term factor; (ST), structural factor. ^∗∗^P = 0.05, ^∗∗∗^P = 0.01.*

Next, three exploratory models of GDP were proposed because three variables representative of GDP were finally significant for the definitive models: GDP_P (Model 1), GDP_PCP (Model 2), and GDP_PC_N (Model 3) (Table 4). Furthermore, considering that the aim of this empirical study is to demonstrate which factors explain a possible secular stagnation of the economy, GDP is the most appropriate endogenous variable.

Based on an analysis of these estimates of the most representative models, the following can be concluded:

• Model 1: $\hat{\mathrm{GDP}\,\_\,P}$ = 6.488 − (0.167 ^∗^ P65_P) − (0.263 ^∗^ UNE) + (1.28 ^∗^ TFPO): The percentage of annual GDP growth is directly explained, to a greater or lesser extent, by the total productivity growth of the original factors,^14^ directly, and indirectly by the unemployment rate and the percentage of the population aged 65 years or older.
• Model 2: $\hat{\mathrm{GDP}\,\_\,P\,\_\,\mathrm{PCP}}$ = 6.316 + (1.63*e* − 07 ^∗^ TD) − (0.168 ^∗^ UNE) − (0.255 ^∗^ P65_P) + (1.244 ^∗^ TFPA): The second model, the percentage growth of annual GDP per capita is explained, gradually from a greater to a lesser extent, by the adjusted TFP growth, directly, and indirectly by the percentage of the population aged 65 years or over and the unemployment rate. Total debt had a positive impact.
• Model 3: $\hat{\mathrm{GDP}\,\_\,\mathrm{PC}\,\_\,N}$ = 82772.01 + (30.846 ^∗^ ST_P) − (71329 ^∗^ CTFP) − (499.877 ^∗^ UNE): In the third model, the total value of stocks traded (as a percentage of GDP) has a direct impact on changes in nominal GDP per capita and counteracts the negative effect exerted by unemployment and TFP (at current prices).

Additionally, the interpretation of the standardized coefficients^15^ obtained in three models (Table 5) makes it possible to conclude which structural and short-term factors explain the behavior of GDP (and, consequently the economic secularization of developed economies) for the established period to the greatest extent. The interpretation is simple: the higher the value of standardized coefficients, the bigger the effect of the factor on GDP. It can thus be concluded from these models that the short-term and structural factors explaining the economic secularization of developed economies could be summarized, in order of relevance (^∗^), as follows (Table 6).

**TABLE 6:** Explanatory power of factors with regard to secular stagnation.

**Variable**	**Model 1**	**Model 2**	**Model 3**
	
	**GDP_P**	**GDP_PCP**	**GDP_PC_N**
TFPO	ST^∗∗∗^		
TFPA		ST^****^	
CTFP			ST^∗∗∗^
UNE	ST^∗∗^	ST^∗∗^	ST^∗∗^
P65_P	ST^∗^	ST^∗∗∗^	
TD		SH^∗^	
ST_P			SH^∗^

ST, structural factor; SH, short-term factor. The number of asterisks (^*^) before a structural (ST) or short-term (SH) factor identifies the degree of relevance in explaining the endogenous variable of each model: one asterisk (^*^) means the minimum degree, two asterisks (^**^) mean a greater degree, three asterisks, (^***^) mean a greater degree, and four asterisks (^****^) mean the maximum degree.

If these models are considered as a whole, clear conclusions can be drawn:

(1) Except for the income and wealth inequality factor (*GINI*), the other indicators (financial cycle, excessive debt, demographic characteristics, fall in TFP, and other factors depressing aggregate demand such as unemployment) seem to significantly explain secular stagnation in advanced economies.
(2) TFP is the factor that most positively affects GDP growth, except in Model 3, where the endogenous variable to be explained is not the growth rate but the nominal value of GDP per capita.
(3) The structural factors explain secular stagnation to a greater extent than the short-term ones. Two of the structural factors are of particular importance: the unemployment rate (UNE) and the aging of the population (P65_P).

### Economic Policies to Combat Secular Stagnation

The econometric panel-data study performed indicates the influence of each explanatory factor with regard to secular stagnation in recent years for the selected sample of countries. Based on these findings, some proposals can be made to enable the government intervention strategies adopted since the onset of the financial crisis by the main developed economies to be reoriented toward the pursuit of sustained growth, job creation, and poverty reduction.

The worsening of the financial crisis in 2008 included the fall of large financial institutions in the United States and Europe, increased government support of the financial system, and an increasingly clear reflection of the turbulence of the financial markets in real economic activity. This situation prompted the G20 leaders to meet for a summit in Washington, held on November 15, to analyze the situation jointly, testimony to the international cooperation effort to implement mechanisms to emerge from the crisis.

In this first phase of the crisis (2008–2009) the G20 action plan was based mainly on the following measures (G20, 2008):

(a) Implementation of *expansionary macroeconomic* (*monetary and fiscal*) *policies* to rapidly stimulate domestic demand:
  • At the *monetary level*, the central banks of the main economies slashed interest rates to encourage household and business consumption and investment in order to boost economic activity and inflation. Additionally, some central banks, especially the U.S. Federal Reserve (Fed), implemented *unconventional monetary policy* instruments (Boards of Governors of the Federal Reserve System, 2019): forward guidance *strategies*, indicating the orientation of monetary policy with respect to the future evolution of official interest rates, conditional on the prospects for price stability; and quantitative easing (QE), i.e., purchase of medium- and long-term bonds, making it possible to raise their prices and, thus, reduce their market interest rates.
  • At the *fiscal level*, discretionary measures were adopted to stimulate the economy, mainly based on increased public spending, tax cuts, and aid for the groups and economic sectors hit hardest by the crisis.
(b) Approval of programs to bail out the financial system intended to increase its strength and solvency and thus facilitate the flow of credit to businesses and consumers in order to reactivate the real economy.
(c) Reform of the international financial architecture, with the priority of strengthening the regulation, oversight, and risk management of the international financial system in order to avoid new systemic crises and mitigate their global impact should they occur.

As a result of these actions, world GDP entered into a phase of mild recovery in 2009, with a return to positive growth in the second half of the year that reached a high rate (5.4% in real terms) in 2010. Furthermore, international financial markets stabilized, volatility and risk aversion markedly declined, asset prices rebounded, and some segments of the capital markets gradually reopened (Banco de España, 2017b: 71).

However, in Europe, and especially in the peripheral countries of the euro zone, due to their greater fiscal and financial vulnerability and worse prospects for economic growth, this recovery process was derailed in early 2010 by the emergence of the first episodes of the sovereign debt crisis, caused by the distrust surrounding the state of Greek public finances.

The origin of this new phase of the crisis was the accumulation by these peripheral countries of significant macroeconomic and financial imbalances during the previous period of economic expansion (1996–2006). These imbalances were mainly associated with the high indebtedness of the private sector, soaring home prices, the excessive concentration of credit in the construction sector and real estate development, overdependence on external financing, and the accumulation of competitiveness losses in their economies (Banco de España, 2017a).

This instability in the euro zone’s sovereign debt markets, which resulted in higher yields on the peripheral countries’ public debt and a considerable increase in the risk premium, hampered their capacity for budgetary adjustment and the recovery of economic activity.

In this environment, the economic policies implemented from 2010 on in the United States and Europe became markedly divergent:

(a) In the *United States*, the Fed continued to adopt unconventional monetary stimulus measures, based on the expansion of the QE program. QE2 was announced on November 3, 2010 and lasted until June 2011. It consisted of the purchase of 600 billion dollars in Treasury bonds, with the aim of keeping interest rates low, making real estate more affordable, and slightly increasing inflation. New fiscal stimulus measures were also implemented in the area of fiscal policy.
  The worsening of the economic crisis in 2012 and 2013 led to the approval of two more programs in the area of monetary policy: *Operation Twist*, an extension of QE2, consisting of the purchase of Treasury notes to influence the economy’s long-term interest rates; and *QE3*, consisting of new purchases of mortgage-backed securities and Treasury bonds.
  However, there was an important change in the direction of *fiscal policy* strategy during this period, basically geared toward reducing the high levels of public deficit and public debt generated by the weakness of real economic activity.
(b) In the *euro zone*, especially since mid-2012, due to the serious relapse of economic activity, an *expansive monetary policy* has continued to be implemented. This policy has mainly been based on reductions in the official interest rates by the European Central Bank, auctions of long-term loans to credit institutions, and reductions in the cash ratio (European Central Bank, 2018).
  However, *fiscal policy* took a clearly contractive turn beginning in 2010, due to the growing instability and uncertainty in sovereign debt markets. During this period, the main priority of government budgetary strategies, especially those of governments that had required external financial assistance, given the explicit conditionality of those programs, was to correct the heavy imbalances recorded in the public accounts (Costas and Arias, 2015). In addition to fiscal consolidation measures aimed at reducing the public deficit and redirecting public debt toward a sustainable path, multiple *structural reform programs* were also implemented covering many areas, including the labor and product market and the public and financial sectors.

In short, this package of fiscal austerity and reform measures sought to achieve more competitive and efficient economies in order to boost sustained growth and job creation, sound public finances geared toward sustainability, a less indebted private sector, and a banking system with a greater capacity to provide credit to the real economy and support recovery (Montoriol-Garriga, 2015; Rosnick and Weisbrot, 2015).

In addition, from 2010 onward, in order to ensure the stability of the European Economic and Monetary Union, an in-depth reform of *European economic governance* was carried out, based mainly on initiatives aimed at strengthening the surveillance of budgetary policies. The main objectives were (a) to achieve an appropriate balance between the sustainability of public accounts and the need to maintain the stabilization function of fiscal policy, (b) to reform the structure of financial supervision in the Union, and (c) to create a permanent mechanism for safeguarding financial stability of the Eurozone (European Stability Mechanism) (Subdirección General de Economía Internacional, 2012; Eur-Lex, 2014; European Commission, 2019; Callejas et al., 2017; Ministerio de Economía Industria y Competitividad, 2019).

All the measures implemented since 2010 allowed for increased deleveraging of the private sector, improved labor markets, increased confidence, and reduced instability in financial markets, which translated to a significant change in growth dynamics by the end of 2013.

Thus, in 2014, the world economy embarked on a path of gradual recovery, with the developed economies receiving a particular boost, thanks to the recent strong performance of international trade, linked to the greater strength of investment (European Central Bank, 2015–2018).

This favorable outlook is also supported by three fundamental factors (Banco de España, 2018):

(a) In contrast to the normalization process followed by the Fed’s monetary policy, the European Economic and Monetary Union has maintained an expansive orientation in this area. Indeed, since spring 2014, the Governing Council of the European Central Bank has been deploying a broad package of measures to avoid the risks to real economic activity arising from the prolongation over time of an excessively low inflation rate.
These actions focus on four basic strategic lines (Banco de España, 2016): the establishment of a negative interest rate for the deposit facility^16^; the communication and publicity policy for future monetary policy orientations; the implementation of specific programs aimed at favoring the proper functioning of transmission mechanisms through banking intermediaries and credit supply; and the implementation of a QE program, consisting of the large-scale purchase of private and public assets.
(b) The approval of fiscal expansion plans in the United States, which include a tax cut and increased infrastructure spending.
(c) Important advances in the institutional design of the European Economic and Monetary Union with the creation of the European Banking Union, which emerged as a step toward financial integration, i.e., toward the single market in financial services, and, in short, toward perfecting the construction of the euro, by restoring the proper functioning of euro zone monetary policy and confidence in the European banking sector (European Central Bank, 2019).

However, despite recent improvements in the international macroeconomic situation, there are still major pockets of risk, especially in the euro zone economies. This risk is mainly associated with uncertainty about the economic policies implemented in the United States and the process of the United Kingdom’s exit from the European Union, in a context marked by persistently high unemployment in some countries (19% in Greece and 15.3% in Spain in 2018), low growth rates (1.9% in the eurozone in 2018), weaknesses in some financial institutions, and an expected rate of medium-term inflation <2%, despite the recent upturn in energy prices (1.8% in the euro zone in 2018). In addition, population aging is more pronounced in European economies, and productivity growth and fiscal consolidation are progressing at a much slower pace than in the United States (Crafts, 2014: 93).

For this reason, and especially as monetary policies are brought back to normal, a process that, as noted, has already begun in the United States, since it is in a more advanced stage of the economic cycle, it is necessary to consider the role that a *more active fiscal policy* could play in reactivating economic activity within the European Economic and Monetary Union (Wolff, 2014: 149).

In this regard, in keeping with Hansen’s initial approach, *increased public investment* in the countries at the heart of the eurozone, which now have more budgetary room for maneuver, could have positive effects for all Member States. Additionally, although peripheral countries must continue with *fiscal consolidation policies* to ensure the medium- and long-term sustainability of public accounts, they can also adopt *more structural measures* aimed at rebalancing the tax burden toward less distorting figures for private activity and, in particular, to enhance public-sector efficiency and synergy with the private sector (by better matching wages to the sector’s productivity, modernizing government through the incorporation of new digital technologies, and increasing collection efficiency) (Aspachs et al., 2016; Banco de España, 2017c).

Furthermore, given the importance of structural factors in explaining the sluggishness of global economic activity in recent years, as seen in the empirical analysis above, to promote sustained and sustainable long-term growth, it is essential to continue implementing *structural reform policies* in different economic sectors, aimed at boosting productivity growth and adequately complementing short-term stabilization policies (monetary and fiscal measures) (Jimeno et al., 2014).

In this regard, it is worth highlighting the need to continue making progress on meeting the different objectives established in the European Union’s growth and employment agenda for the current decade, i.e., the *Europe 2020 Strategy*. The strategy highlights “intelligent, sustainable and inclusive growth as a way of overcoming the structural deficiencies of the European economy, improving its competitiveness and productivity and sustaining a sustainable social market economy” (European Commission, 2019).

In particular, the action lines of the Europe 2020 Strategy focus on five main areas (Eur-Lex, 2017):

(a) *Employment*: achieve a minimum employment rate of 75% for the population aged 20–64 years.
(b) *Research and development (R&D)*: invest 3% of GDP in R&D.
(c) *Climate change and energy:* reduce greenhouse gas emissions by at least 20%, increasing the share of renewable energy sources in our final energy consumption to 20% and energy efficiency by 20%.
(d) *Education:* reduce the drop-out rate to <10% and increase the rate of higher-education qualifications to at least 40%.
(e) *Poverty and social exclusion:* reduce the number of people living below the poverty line or at risk of social exclusion by 20 million.

At the same time, in order to continue boosting the real economy, the *United States* should implement a *fiscal policy* aimed at promoting public infrastructure, education, and R&D with a view to improving productivity, boosting potential economic growth, increasing the employability of groups that have lost their jobs, and reducing possible sources of financial instability. Furthermore, *redistributive policies* aimed at reducing inequality in income and wealth distribution (Berganza and L’Hotellerie-Fallois, 2017), coupled with *structural reforms* to improve the social safety net and human capital formation, would be needed to reduce the secular increase in the number of unemployed adults over the last four decades (Glaeser, 2014).

Table 7 offers a summary of the most relevant economic policy measures adopted at the international level since the onset of the economic and financial crisis, as well as the main proposals to reactivate real economic activity.

**TABLE 7:** Economic policy measures to combat secular stagnation.

**Time scope**	**Economic policy measures and recommendations**
1st phase of the crisis (2008–2009)	• Expansive macroeconomic (monetary and fiscal) policies to stimulate aggregate demand
	• Financial system rescue programs
	• Reform of the international financial architecture
2nd phase of the	(a) United States
crisis (2010–2013)	2010–2011:
	• Unconventional monetary stimulus measures
	• Budgetary stimulus measures.
	2012–2013:
	• New monetary stimulus programs
	• Fiscal policy aimed at reducing high levels of indebtedness
	(b) Euro zone
	• Expansive monetary policy
	• Contractive fiscal policy aimed at correcting the strong imbalances in public accounts (fiscal consolidation)
	• Structural reform programs
	• Reform of European economic governance (six-pack, two-pack, Budgetary Pact, reform of financial supervision, and creation of the ESM)
Recovery phase	(a) United States
(2014–2017)	• Monetary policy normalization process
	• Fiscal expansion plans
	(b) Euro zone
	• Expansive orientation of monetary policy
	• Advances in the institutional design of the EMU: creation of the European Banking Union
Future prospects	(a) More active fiscal policy
(euro zone)	• Increases in public investment at the heart of the euro zone
	• Fiscal consolidation policies and more structural measures on the periphery (rebalancing the tax burden and improving public-sector efficiency)
	(b) Structural reform policies: Europe 2020 Strategy (smart, sustainable, and inclusive growth):
	• Jobs and careers
	• Research and development
	• Climate change and energy
	• Education
	• Poverty and social exclusion
Future prospects	(a) Fiscal policy
(United States)	• Public infrastructure
	• Education
	• R&D
	(b) Redistributive policies
	• Combat inequality
	(c) Structural reforms
	• Improving the Social Security network
	• Human capital formation

## Discussion

In addition to the more typically short-term factors associated with the effects of the recent global financial crisis and the previously high levels of public and private debt, the chronic insufficiency of aggregate demand, which would support the secular stagnation hypothesis, can be explained, mainly in the central economies, by long-term or structural changes in recent decades. These changes include: the rapid deceleration of demographic growth and gradual aging of the population; the fall in TFP; growing inequality in income and wealth distribution; the gradual exhaustion of the globalization process; and the consolidation of new labor models that undermine employment stability, among others.

The empirical analysis carried out in the present research quantified the impact of these short-term and structural factors on economic growth by means of an econometric panel-data study. The sample consisted of the 12 countries around the world that the World Bank considered powers in 2017, as they had the highest level of GDP per capita over a 20-year time horizon (1998–2017).

Tests were carried out, and three final valid models were selected, all of which explain economic growth.

Of the two groups of factors analyzed, the structural ones (demographic, TFP, income and wealth distribution, and employment) had a stronger influence on secular stagnation than the short-term ones (financial cycle and excessive accumulated public and private debt).

More specifically, in the three proposed models, TFP proved to be the most significant variable and the one to most greatly affect the evolution of growth in the economies under consideration. In contrast, the unequal distribution of income (as measured by the Gini index) did not seem to influence secular stagnation, as it was not significant in any of the proposed models.

Although the cyclical factors, quantified in the behavior of financial markets and in excessive public and private debt, did influence economic growth in two of the three proposed models, their influence was much smaller.

Consequently, given the importance of structural factors in explaining the sluggishness of economic activity in recent years, to promote sustained and sustainable growth in the long term, economic stabilization policies (monetary and fiscal) must be adequately complemented by structural reform policies.

In view of the difficulty of adopting policies to increase the birth rate and combat the problem of the rapid slowdown in the growth of the world population and its gradual aging, these structural measures should be aimed, in particular, at improving the employability of the most disadvantaged groups in the labor market, promoting R&D, curbing climate change and encouraging energy savings, implementing training and education programs, and combating poverty and social exclusion.

Certainly, environmental factors and economic policies are determinant; human motivation also plays a critical role in the entrepreneurial process (Shane et al., 2003).

Measures and motivational factors must be offered to support the behavior of people with an entrepreneurial drive (Ajzen, 1991).

In short, all these policies will ultimately translate to improved productivity and competitiveness innovation and entrepreneurship, as key factors in promoting job creation, raising the potential growth of economies and economic and social cohesion. Consumers would thus perceive favorable expectations and secular stagnation would be avoided.

## Data Availability Statement

All datasets generated for this study are included in the article/Supplementary Material.

## Author Contributions

All authors listed have made a substantial, direct and intellectual contribution to the work, and approved it for publication.

## Conflict of Interest

The authors declare that the research was conducted in the absence of any commercial or financial relationships that could be construed as a potential conflict of interest.

## Footnotes

1 For more detailed information, see Hansen (1939).

2 Specifically, the war industry became a dynamizing factor in the post-war American economy, with large companies such as General Electric and Westinghouse in atomic power plants, IBM in computers, and Boeing in civil aviation and space programs. This large industrial growth required more labor force, which contributed to the creation of millions of jobs and a significant increase in the volume of production. In addition, after World War II, President Harry S. Truman promoted a program aimed at the gradual suppression of state control in the economy, which established an ambitious reform of social expenditure to make it more efficient and less costly. In this way, a welfare state was built that made it possible to reduce social inequalities, thanks to the approval of a plan for social housing for families with lower incomes, the control of rents and agricultural subsidies, and improvements in civil rights, employment, education, public health, and social benefits. For a detailed analysis of the role of the public sector in economic activity and its contribution to growth and technological innovation, see Galbraith (1984) and Mazzucato (2014).

3 For a detailed study, see Summers (2013).

4 According to the Spanish National Statistics Institute (INE), TFP is the difference between the production growth rate and the average growth rate of the factors used to obtain it, which are weighted by the share of national income that each factor accounts for. In other words, the sum of the contributions of productive factors (labor and capital) and TFP is the same as the rate of change of GDP.

5 In addition to the paralysis in technological innovation, Gordon (2014) argues that economic growth will continue to be slow over the next 25–40 years because of four “headwinds”: an aging population; lower secondary school and university completion rates, coupled with greater difficulties for university graduates to find jobs suited to their education; inequality in income distribution; and high public indebtedness.

6 In fact, some authors, such as Mokyr (2014), professor of economics and history at Northwestern University, argue that technological progress, particularly in areas such as computing, materials, and genetic engineering, has significant indirect effects on productivity growth via the tools that scientific research provides.

7 The degree of inequality in a country is usually determined based on the available monetary income. Specifically, the distribution of this income is usually measured by defining population segments, starting with those with the lowest income, and determining what proportion of income corresponds to each one. To this end, the most commonly used indicator is the so-called Gini coefficient, which measures income inequality among the citizens of a given territory, usually a country. This index ranges from 0 to 1, where 0 is the maximum equality (all citizens have the same income) and 1 is the maximum inequality (all income is owned by a single individual). Thus, the closer the coefficient is to 0, the less inequality there will be; conversely, the closer it is to 1, the more the inequality will grow (Torres, 2016).

8 The eighth round of multilateral trade negotiations (Uruguay Round) resulted in the largest reform of the world trading system since the creation of the General Agreement on Tariffs and Trade (GATT) in 1948. It began in Punta del Este (Uruguay) in 1986 and concluded in Marrakech (Morocco) on December 15, 1993. Despite the challenges to be overcome, the Uruguay Round made significant efforts to establish a more open and transparent global trading system, both because of the large number of participants (123) and because of the importance of the issues addressed: the negotiation agenda on the access of goods to international markets; the first steps in the liberalization of international trade in services; the drafting of principles to guarantee the intellectual property incorporated in the goods and services exchanged and foreign investments in any country; and, finally, institutional agreements to replace the GATT with the World Trade Organization (WTO) (Zapata and Gabriele, 1994).

9 For detailed information, see Adhikari and Yang (2002).

10 The econometric modeling software used has been Stata.11 (Statacorp, 2009).

11 The 12 selected countries are those that the World Bank considered global powers in 2017, as they had registered the highest level of GDP per capita over the period: Brazil, China, Canada, France, Germany, India, Italy, Japan, Korea, Spain, the United Kingdom, and the United States.

12 The Hausman test is used to determine the most appropriate estimation method – FEs or random effects (REs) – in order to obtain the most robust parameters in each case. In this analysis no test has been made because of an algebraical problem. However, the characteristics of the sample mentioned determine that the FEs be the most appropriate method.

13 The value of the stocks traded is the total number of shares traded, both domestic and foreign, multiplied by their respective matching prices. Only part of the transaction is considered. Companies admitted to listing and admitted to trading are included in the data. Data are year-end values.

14 The Total Economy Database (TED) is structured in two parts: TED1, containing output, labor, and productivity data, and TED2, containing growth data. This latter part, in turn, includes two versions of the data: (1) *The Conference Board* (*TCB*) *Adjusted:* For the calculation of the adjusted version of data, GDP growth rates are adjusted for several countries. More specifically, GDP deflators are used for three countries with significant ICT production and trade: China, Japan, and the United States. This translates to higher real GDP growth rates for these countries compared to the original data version. (2) *The Conference Board* (*TCB*) *Original:* Based on official GDP data for the period after 1990 that do not reflect the rapid declines in ICT goods.

15 When measurement scale of the independent variables is different (Tables 2, 3), standardized coefficients must be used to interpret and compare their effects on your dependent variable. The standardized coefficients obtained in the three models are not an output of Stata software, but have been calculated directly from the unstandardized coefficients along with the standard deviations of the variables involved: ${\hat{\beta }}_{J}^{{}^{*}}={\hat{\beta }}_{J}*\frac{\mathrm{SD}\,\left(X_{j}\right)}{\mathrm{SD}\,\left(Y\right)}$

16 The Eurosystem’s standing facility, which counterparts (the euro zone credit institutions with which the Eurosystem conducts certain monetary policy operations) may use to make overnight deposits with a national central bank remunerated at a pre-specified interest rate (Banco de España, 2019).
