# Supplementary material for: Consumer Motivation in Developed Economies With Secular Stagnation
Source: Front Psychol. 2019 Dec 2;10:2697. doi: 10.3389/fpsyg.2019.02697 (PMC6900960; doi:10.3389/fpsyg.2019.02697)
Supplement: Supplementary file 1 [file Data_Sheet_1.PDF]

Supplementary Material

1 Supplementary Figures and Tables

1.1 Supplementary Table 1. Endogenous and exogenous variables used in the models

**EXOGENOUS VARIABLES**				
**Short-term factors**				
**Variable**	**Acronym**	**Database**	**Definition**	**Factor**
Stocks traded, total value (% of GDP)	*ST_P*	World Federation of Exchanges database.	The value of the shares traded is the total number of shares traded, both domestic and foreign, multiplied by their respective matching prices. Only part of the transaction is considered. Companies admitted to listing and admitted to trading are included in the data. Dates are year-end values.	Financial Cycle
Stocks traded, total value (US$ at current prices)	*ST*	World Federation of Exchanges database.	The value of the shares traded is the total number of shares traded, both domestic and foreign, multiplied by their respective matching prices. Only part of the transaction is considered. Companies admitted to listing and admitted to trading are included in the data. Data are year-end values converted to US dollars using year-end foreign exchange rates.	
Total Debt	*TD*	Datosmacro.com	Total debt (current euros)	Debt
Total debt (per capita)	*TD_PC*		Total debt per capita (current euros)	
**Structural factors**				
**Variable**	**Acronym**	**Database**	**Definition**	**Factor**
Population aged 65 and over (total)	*P65*	World Bank. Based on the United Nations Population Division's World Population Prospects age/sex distribution (updated in 2017).	Population is based on the definition of de facto population, which refers to the national legal status of citizenship.	Demographics
Population aged 65 and over (% of total)	*P65_P*			

Population aged 0-14 (% of total)	*P14*		Population between the ages of 0 and 14 as a percentage of the total population. Population is based on the definition of de facto population.	
Population growth (annual %)	*PG_P*	Sources: (1) United Nations Population Division, World Population Prospects: 2017 Revision; (2) Census reports and other statistical publications of national statistics bureaus; (3) Eurostat: Demographic Statistics; (4) United Nations Statistical Division: Population and Vital Statistics Report (various years); (5) U.S. Census Bureau: International Database; and (6) Secretariat of the Pacific Community: Statistics and Demography Programme.	Annual population growth rate for year t is the exponential rate of population growth at mid-year from year t-1 to t, expressed as a percentage. Population is based on the definition of de facto population, which refers to the national legal status of citizenship.	
Unemployment, total (% of total workforce)	*UNE*	International Labour Organization, ILOSTAT database (updated 2017).	Unemployment refers to the percentage of the workforce that does not have work, but is available to seek employment.	Aggregate demand
Gini Index	*GINI*	World Bank, Development Research Group. The data are based on primary household survey data obtained from government statistical agencies and country departments of the World Bank.	The Gini coefficient measures the extent to which income distribution (or, in some cases, consumption expenditure) among individuals or households within an economy deviates from a perfectly equal distribution. The Gini coefficient measures the area between the Lorenz curve and a hypothetical line of absolute equality, expressed as a percentage of the maximum area below the line. Therefore, a Gini coefficient of 0 represents perfect equality, while a Gini coefficient of 100 implies perfect inequality.	Inequality of income
Total Factor Productivity (TFP) level at current PPPs (USA=1).	*CTFP*	Penn World Table, version 9.0 (Feenstra et al., 2015)) www.ggdc.net/pwt	TFP at current prices (USA=1). GDP at current prices, capital and TFP.	Total factor productivity
TFP at constant national prices (2011=1)	*RTFPNA*		TFP at constant national prices (2011=1) Variables based on national accounts.	
TFP growth (adjusted)	*TFPA*	THE CONFERENCE BOARD. Total Economy Database	Description of variables<br><br>Growth rates	

TFP growth (original)	*TFPO*		1. GDP growth (log)<br>Contributions to GDP growth<br>2. Contribution by amount of labor. Contribution of labor quantity to GDP growth (7 = 2 * 13)<br>3. Contribution of labor quality. Contribution of quality of work to GDP growth (8 = 3 * 13)<br>4. Total capital contribution. Contribution of total capital services to GDP growth (9 = 4 * 14)<br>TFP growth (12 = 1-2-3-4)	
**ENDOGENOUS VARIABLES**				
**Variable**	**Acronym**	**Database**	**Definition**	
GDP growth (annual %)	*GDP_P*	World Bank national accounts data and OECD national accounts data.	Annual percentage growth rate of GDP at market prices based on constant local currency. Aggregates are based on 2010 U.S. dollars. GDP is the sum of the gross value added of all producers in the economy plus any product taxes and minus any subsidies not included in the value of products. It is calculated without depreciation of manufactured assets or for depletion and degradation of natural resources.	
GDP growth per capita (annual %)	*GDP_PCP*			
GDP per capita (current US dollars)	*GDP_PC_N*		GDP per capita is the GDP divided by the population at mid-year. GDP is the sum of the gross value added of all producers in the economy plus any product taxes and minus any subsidies not included in the value of products. It is calculated without depreciation of manufactured assets or for depletion and degradation of natural resources. Data are in current US dollars.	
GDP per capita (constant local currency)	*GDP_PC_R*		GDP per capita is the GDP divided by the population at mid-year. GDP at purchaser prices is the sum of the gross value added of all producers in the economy plus any product and minus any subsidies not included in the value of products. It is calculated without depreciation of manufactured assets or for depletion and degradation of natural resources. The data are in constant local currency.	

1.2 Supplementary Dataset 1. Correlation matrix

	st_p	st	td	td_pc	pg_p	gdp_p	gdp_pcp	une
**st_p**	1							
**st**	0,806	1						
**td**	0,585	0,866	1					
**td_pc**	-0,057	0,025	0,032	1				
**pg_p**	0,112	0,060	-0,123	-0,022	1			
**gdp_p**	0,101	0,072	-0,050	0,391	0,189	1		
**gdp_pcp**	0,077	0,060	-0,024	0,402	-0,031	0,976	1	
**une**	-0,187	-0,164	-0,146	-0,171	-0,161	-0,436	-0,409	1
**gini**	-0,258	0,055	-0,022	0,047	0,367	0,215	0,137	-0,004
**gdp_pc_n**	0,415	0,311	0,400	-0,294	-0,321	-0,386	-0,321	-0,026
**gdp_pc_r**	0,232	-0,050	-0,125	-0,052	-0,060	0,185	0,201	-0,273
**p65**	0,177	0,372	0,375	0,859	-0,059	0,423	0,444	-0,241
**p65_p**	0,073	-0,068	0,115	-0,222	-0,454	-0,509	-0,417	0,246
**p14**	-0,184	0,089	-0,020	0,086	0,418	0,335	0,248	-0,196
**ctfp**	0,358	0,287	0,312	-0,333	-0,257	-0,291	-0,239	0,085
**rtfpna**	-0,031	-0,119	-0,058	0,008	0,057	-0,098	-0,113	0,048
**tfpa**	0,126	0,154	0,113	0,358	-0,132	0,779	0,822	-0,197
**tfpo**	0,135	0,123	0,076	0,333	-0,141	0,784	0,830	-0,212
	**gini**	**gdp_pc_n**	**gdp_pc_r**	**p65**	**p65_p**	**p14**	**ctfp**	**rtfpna**
**gini**	1							
**gdp_pc_n**	-0,703	1						
**gdp_pc_r**	-0,151	-0,139	1					
**p65**	0,139	-0,223	-0,120	1				
**p65_p**	-0,802	0,761	-0,198	-0,254	1			
**p14**	0,829	-0,630	-0,081	0,149	-0,883	1		
**ctfp**	-0,712	0,778	-0,210	-0,255	0,708	-0,579	1	
**rtfpna**	-0,375	0,338	-0,304	-0,136	0,443	-0,402	0,433	1
**tfpa**	0,027	-0,133	0,073	0,432	-0,192	0,119	-0,069	-0,174
**tfpo**	-0,015	-0,135	0,180	0,378	-0,197	0,097	-0,073	-0,183

1.3 Supplementary Dataset 2. Results of econometric models: panel-data estimation

Model 1. $GDP\_P = f(+TFPO, -UNE, -P65\_P\ )$

```
. xtreg  gdp_p  p65_p une tfpo,fe

Fixed-effects (within) regression               Number of obs      =       228
Group variable: ip                              Number of groups   =        12

R-sq:  within  = 0.7382                         Obs per group: min =        19
       between = 0.8257                                        avg =      19.0
       overall = 0.7788                                        max =        19

                                                F(3,213)           =    200.18
corr(u_i, Xb)  = -0.4178                        Prob > F           =    0.0000

------------------------------------------------------------------------------
       gdp_p |      Coef.   Std. Err.      t    P>|t|     [95% Conf. Interval]
-------------+----------------------------------------------------------------
       p65_p |  -.1669301   .0509322    -3.28   0.001    -.2673258   -.0665345
         une |  -.2632166   .0361817    -7.27   0.000    -.3345367   -.1918965
        tfpo |   1.280557   .0552099    23.19   0.000     1.171729    1.389385
       _cons |   6.488171   .7678111     8.45   0.000     4.974689    8.001652
-------------+----------------------------------------------------------------
     sigma_u |  1.2716895
     sigma_e |  1.1536485
         rho |  .54855492   (fraction of variance due to u_i)
------------------------------------------------------------------------------
F test that all u_i=0:     F(11, 213) =    10.45             Prob > F = 0.0000
```

Model 2. $GDP\_P\_PCP = f(+TFPA, -P65\_P, -UNE, +TD)$

```
. xtreg gdp_pcp td une p65_p tfpa,fe

Fixed-effects (within) regression               Number of obs      =       219
Group variable: ip                              Number of groups   =        12

R-sq:  within  = 0.6817                         Obs per group: min =        17
       between = 0.8111                                        avg =      18.3
       overall = 0.7337                                        max =        19

                                                F(4,203)           =    108.71
corr(u_i, Xb)  = -0.5413                        Prob > F           =    0.0000

------------------------------------------------------------------------------
     gdp_pcp |      Coef.   Std. Err.      t    P>|t|     [95% Conf. Interval]
-------------+----------------------------------------------------------------
          td |   1.63e-07   7.33e-08     2.23   0.027     1.87e-08    3.08e-07
         une |  -.1676358    .038941    -4.30   0.000    -.2444165    -.090855
       p65_p |  -.2553896   .0663034    -3.85   0.000    -.3861212    -.124658
        tfpa |   1.244584   .0626603    19.86   0.000     1.121036    1.368133
       _cons |   6.316383   .9270275     6.81   0.000     4.488545    8.144221
-------------+----------------------------------------------------------------
     sigma_u |  1.3973022
     sigma_e |  1.2267496
         rho |  .56472249   (fraction of variance due to u_i)
------------------------------------------------------------------------------
F test that all u_i=0:     F(11, 203) =     5.22             Prob > F = 0.0000
```

Model 3. $GDP\_PC\_N = f(+ST\_P, -UNE, -CTFP)$

```
. xtreg  gdp_pc_n st_p ctfp une,fe

Fixed-effects (within) regression               Number of obs      =       196
Group variable: ip                              Number of groups   =        12

R-sq:  within  = 0.3626                         Obs per group: min =        12
       between = 0.7828                                        avg =      16.3
       overall = 0.4869                                        max =        17

                                                F(3,181)           =     34.33
corr(u_i, Xb)  = -0.9399                        Prob > F           =    0.0000

------------------------------------------------------------------------------
    gdp_pc_n |      Coef.   Std. Err.      t    P>|t|     [95% Conf. Interval]
-------------+----------------------------------------------------------------
        st_p |   30.84566   11.77316     2.62   0.010     7.615367    54.07596
        ctfp |  -71329.04   7158.865    -9.96   0.000    -85454.61   -57203.47
         une |  -499.8768    192.802    -2.59   0.010    -880.3054   -119.4482
       _cons |   82772.01    5934.73    13.95   0.000     71061.86    94482.17
-------------+----------------------------------------------------------------
     sigma_u |  31337.329
     sigma_e |  5611.3454
         rho |  .96893269   (fraction of variance due to u_i)
------------------------------------------------------------------------------
F test that all u_i=0:     F(11, 181) =    43.11             Prob > F = 0.0000
```
